# Long-Term Disability Outcomes for Migrants (and Non-migrants) 12 Years Post-injury: Results from the Prospective Outcomes of Injury Study in New Zealand

**DOI:** 10.1007/s10903-023-01526-w

**Published:** 2023-08-05

**Authors:** A. Anglemyer, E. H. Wyeth, S. Derrett

**Affiliations:** Ngāi Tahu Māori Health Research Unit, Division of Health Sciences, University of Otago, Dunedin, New Zealand

**Keywords:** Injury, Migrant, Long-term, Longitudinal, Cohort

## Abstract

To understand, and identify predictors of, long-term post-injury (i.e. 12 years post-injury) disability outcomes for migrants and non-migrants. This 12-year longitudinal study followed participants with entitlement claim injuries registered with New Zealand’s universal no-fault injury insurer between 2007 and 2009. Information was collected about migrant status, other sociodemographic, health and disability characteristics, and injury characteristics. Disability outcome information was collected 12 years later. Of 1543 people interviewed 12 years post-injury, 1497 had disability and migrant status data available; 20% were migrants (n = 301). Migrants reporting inadequate pre-injury household income or those who perceived their injury as a threat to life at the time of injury were more likely to experience disability 12 years post-injury (aRR 2.08; 95% CI 1.09–4.03, aRR 2.93; 95%CI 1.17–6.69, respectively). Hospitalised injured migrants were significantly less likely to have long-term disability (aRR 0.18; 95%CI 0.04–0.55) than those not hospitalised. We found sociodemographic and injury-related characteristics were independently associated with long-term disability among migrants. We highlight that some characteristics, ascertained early in the injury pathway, predict risk of long-term disability. Early post-injury interventions focused on improving disability outcomes for migrants may also have long-term impacts.

**Supplementary Information:**

The online version contains supplementary material available at 10.1007/s10903-023-01526-w.

## Introduction

Internationally, researchers have investigated outcomes among migrant populations such as wellbeing, health, and job satisfaction [1]. A recent systematic review noted that migrants can experience a range of adverse experiences, including discrimination and marginalisation [1]. The importance of understanding the risks for migrants associated with injury and long-term disability appears to have received less attention to date [2]. Where research into injury-related disability has been undertaken, it tends to have focused on migrants injured in the workplace [3–5].

In New Zealand (NZ), it is estimated that over a third of disability occurs as a consequence of injury [6]. Additionally, it is estimated that nearly 8% of health loss is from injury [7]. NZ has a unique universal no-fault injury insurance scheme operated by the Accident Compensation Corporation (ACC), a Crown entity, to support injured people. In addition to earnings-related compensation if a person requires time off work due to an injury (up to 80% of pre-injury income), ACC also provides subsidies for transport assistance, accommodation, prescriptions, treatments, and rehabilitation for injuries [8].

Importantly, migrants have unique histories and experiences that need to be considered in the context of injury prevention and treatment. Migrants have been found to have higher risks of occupational injuries [9, 10], while also having poorer injury outcomes compared to those born in their country of residence [11]. For example, in the US, migrants’ use of healthcare services is significantly lower than non-migrants’ use [12]. While migrants (i.e., ‘naturalised citizens’, legal residents, and undocumented immigrants) in the US account for approximately 14% of the total population, they utilise less than 10% of all healthcare costs [13].

Additionally, the patterns of migration have been changing over time in NZ. In 2013, migrants comprised approximately 25% of the NZ population, increasing from approximately 20% in 2001 [14]. In 2018, 27% of NZ’s population was born elsewhere [15]. There has been very little immigration since 2020 due to the ongoing COVID-19 pandemic and border closures [16].

In NZ, earlier phases of the Prospective Outcomes of Injury Study (POIS) found migrants had a higher risk of disability than non-migrants three months post-injury, but not by 24 months post-injury [2]. Little is currently known about long-term disability outcomes following injuries among migrants to NZ. Therefore, in this paper we aim to: (1) describe long-term disability outcomes for injured migrants and non-migrants; (2) identify characteristics associated with long-term disability outcomes; (3) consider differences in characteristics between injured migrants and non-migrants; (4) and determine whether the identified predictors of disability differ from our earlier analyses of migrants’ disability outcomes to 24 months [2].

## Methods

### Participants

POIS is 12-year longitudinal cohort study following individuals who had ACC entitlement claims, and were recruited via ACC between December 2007 and June 2009 [17, 18]. Participants were aged 18–64 years at the time of injury. Visitors to NZ at the time of injury and people experiencing self-harm or sexual assault-related injuries were ineligible for inclusion. Injuries were wide-ranging, with a quarter requiring hospitalisation. All participants provided informed consent; ethical approval for POIS was obtained from the NZ Health and Disability Multi-region Ethics Committee (MEC/07/07/093). The recruitment strategy and follow-up rates have been previously described [17].

### Data Collection

Participants self-reported data were collected through structured telephone interviews (89%) or postal questionnaires (11%) [18]. Data were also obtained, with participants’ consent, from ACC’s electronic information about the injury that led to their recruitment to POIS, and from the Ministry of Health about injury-related hospitalisations for the 25% of participants who were hospitalised. Interview questions asked about: pre-injury health characteristics, and injury-related characteristics undertaken 3 months post-injury (on average), and for disability outcomes 12 years after injury. Analyses of 24 month post-injury data in relation to migrants and predictors of disability have been previously described [19].

### Measures

The primary exposure of interest for these analyses was participants’ migrant status. Using the question from the 2006 NZ Census (“Which country were you born in?”) [20], participants were classified as migrants if they were not born in NZ [2].

To measure disability, the WHODAS II was used [21, 22]. This includes six domains of adult functioning (cognition, getting along, self-care, mobility, life activities, and participation) [21, 22]. Specifically, participants rated 12 items from no difficulty (0) to extreme difficulty/cannot do (4), and the cumulative total across all items (48 maximum) was calculated for each participant. Disability scores were dichotomised into “No/Lesser” (< 10) or “Considerable” (≥ 10) disability [19]. If a participant’s response was missing for one item, a score for that item was imputed based on the average of the other 11 items. If more than one item was missing, the participant’s outcome score was not included. Injury severity was measured by the New Injury Severity Score (NISS) [23]; description and categorization of all other characteristics have been presented previously [19].

### Analysis

To describe the relative differences between migrants and non-migrants with regards to pre-injury characteristics and demographics, we employed *X*^2^ tests of independence (Table 1). As several characteristics were found to be significantly different between migrant and non-migrant participants, we analysed migrants and non-migrants separately.

**Table 1:** Select characteristics according to migrant/non-migrant status

	Migrants (n = 301)	Non-migrants (n = 1196)	p-value
	n (%)	n (%)	
Pre-injury sociodemographic characteristics			
Gender			
Male	171 (56.8)	669 (55.9)	0.835
Female	130 (43.2)	527 (44.1)	
Age at time of injury (years)			
18–24	18 (6.0)	129 (10.8)	0.017
25–34	55 (18.3)	220 (18.4)	
35–44	73 (24.3)	280 (23.4)	
45–54	81 (26.9)	354 (29.6)	
55–64	74 (24.6)	213 (17.8)	
Highest education qualification			
Post-secondary school	216 (73.0)	722 (61.1)	< 0.001
Secondary school	66 (22.3)	285 (24.1)	
No formal	14 (4.7)	174 (14.7)	
Paid employment			
Yes	284 (94.7)	1106 (92.5)	0.231
No	16 (5.3)	90 (7.5)	
Household income			
Adequate	192 (64.2)	837 (70.5)	0.041
Inadequate	107 (35.8)	350 (29.5)	
Living arrangements			
With family	225 (75.0)	845 (70.8)	0.332
With non-family	51 (17.0)	228 (19.1)	
Alone	24 (8.0)	120 (10.1)	
Social relationships			
Satisfied	280 (94.0)	1118 (93.9)	0.999
Dissatisfied	18 (6.0)	73 (6.1)	
Sense of community			
Strong	98 (34.9)	341 (29.5)	0.201
In-between	122 (43.4)	528 (45.8)	
Very little	61 (21.7)	285 (24.7)	
Comfort in faith or spiritual beliefs			
Very much/quite a bit	136 (47.4)	331 (28.9)	< 0.001
Somewhat/a little bit	93 (32.4)	405 (35.4)	
Not at all	58 (20.2)	409 (35.7)	
Family involvement			
Very large/large	35 (11.8)	141 (11.8)	0.999
Small/very small	262 (88.2)	1052 (88.2)	
Pre-injury health-related characteristics			
General health			
Not poor health	290 (96.3)	1136 (95.1)	0.462
Poor health	11 (3.7)	58 (4.9)	
Chronic conditions (any)			
No	10 (3.3)	36 (3.0)	0.925
Yes	291 (96.7)	1160 (97.0)	
Disability (WHODAS II)			
No/lesser (0–9)	290 (97.6)	1125 (94.9)	0.058
Considerable (≥ 10)	7 (2.4)	61 (5.1)	
Smoking			
No	245 (81.9)	896 (75.0)	0.014
Yes	54 (18.1)	299 (25.0)	
Regular alcohol use			
No	58 (19.4)	100 (8.4)	< 0.001
Yes	241 (80.6)	1094 (91.6)	
Recreational drug use			
No	274 (91.6)	987 (82.7)	< 0.001
Yes	25 (8.4)	206 (17.3)	
Injury-related characteristics			
Work-related injury			
No	97 (32.3)	375 (31.4)	0.818
Yes	203 (67.7)	818 (68.6)	
Injury severity (NISS)			
1–3	135 (46.2)	446 (38.5)	0.045
4–6	124 (42.5)	579 (50.0)	
≥ 7	33 (11.3)	133 (11.5)	
Hospitalisation			
No	220 (73.1)	911 (76.2)	0.300
Yes	81 (26.9)	285 (23.8)	
Perceived threat to life			
No	261 (88.2)	1068 (90.7)	0.240
Yes	35 (11.8)	110 (9.3)	
Perceived threat of long-term disability			
No	177 (59.2)	711 (60.8)	0.655
Yes	122 (40.8)	458 (39.2)	
Trouble accessing healthcare services			
No	261 (87.9)	1057 (89.0)	0.666
Yes	36 (12.1)	131 (11.0)	

In univariate analyses, we used a modified Poisson regression to estimate the effects of each predictor on disability at 12 years post-injury (Table 2) [24]. In the primary analysis, we used modified Poisson multiple regression to determine predictors of experiencing disability at 12 years post-injury specifically among migrants (Table 3).

**Table 2:** Univariate analyses—associations between selected pre-injury and injury-related characteristics and disability 12 years post-injury according to migrant/non-migrant status

	Migrants (n = 301)		Non-migrants (n = 1196)	
	RR (95% CI)	p-value	RR (95% CI)	p-value
Pre-injury sociodemographic characteristics				
Sex				
Female	Ref		Ref	
Male	0.73 (0.41–1.28)	0.270	0.77 (0.59–1.01)	0.061
Age at time of injury (years)				
18–24	Ref		Ref	
25–34	1.15 (0.28–7.69)	0.866	1.13 (0.62–2.17)	0.693
35–44	1.73 (0.48–10.99)	0.470	1.78 (1.04–3.26)	0.046
45–54	1.89 (0.54–11.91)	0.395	1.82 (1.08–3.30)	0.034
55–64	1.09 (0.28–7.18)	0.908	1.41 (0.79–2.66)	0.263
Highest education qualification				
Post-secondary school	0.67 (0.24–2.79)	0.507	0.55 (0.40–0.77)	< 0.001
Secondary school	0.99 (0.32–4.29)	0.987	0.51 (0.34–0.77)	0.001
No formal	Ref		Ref	
Paid employment				
Yes	0.50 (0.22–1.43)	0.137	0.56 (0.40–0.85)	0.005
No	Ref		Ref	
Household income				
Adequate	Ref		Ref	
Inadequate	2.74 (1.55–4.96)	< 0.001	1.46 (1.10–1.92)	0.008
Living arrangements				
With family	0.46 (0.21–1.13)	0.062	0.92 (0.60–1.48)	0.704
With non-family	0.74 (0.29–2.01)	0.533	1.15 (0.70–1.94)	0.591
Alone	Ref		Ref	
Social relationships				
Satisfied	0.45 (0.21–1.18)	0.067	0.75 (0.47–1.27)	0.245
Dissatisfied	Ref		Ref	
Sense of community				
Strong	0.92 (0.45–1.82)	0.806	1.28 (0.93–1.77)	0.127
In-between	Ref		Ref	
Very little	1.05 (0.47–2.22)	0.896	1.24 (0.88–1.74)	0.213
Comfort in faith or spiritual beliefs				
Very much/quite a bit	2.22 (0.93–6.56)	0.103	1.61 (1.14–2.29)	0.007
Somewhat/a little bit	1.75 (0.67–5.40)	0.285	1.28 (0.90–1.82)	0.167
Not at all	Ref		Ref	
Family involvement				
Very large/large	Ref		Ref	
Small/very small	0.69 (0.34–1.58)	0.327	0.67 (0.48–0.98)	0.033
Pre-injury health-related characteristics				
General health				
Not poor health	Ref		Ref	
Poor health	1.72 (0.42–4.69)	0.363	3.23 (2.15–4.67)	< 0.001
Chronic conditions (any)				
No	Ref		Ref	
Yes	0.81 (0.25–4.94)	0.767	2.16 (0.82–8.74)	0.185
Disability (WHODAS II)				
No/lesser (0–9)	Ref		Ref	
Considerable (≥ 10)	4.93 (1.70–11.34)	< 0.001	2.81 (1.85–4.10)	< 0.001
Smoking				
No	Ref		Ref	
Yes	1.16 (0.55–2.24)	0.669	1.74 (1.31–2.30)	< 0.001
Regular alcohol use				
No	Ref		Ref	
Yes	0.60 (0.33–1.16)	0.108	0.50 (0.35–0.73)	< 0.001
Recreational drug use				
No	Ref		Ref	
Yes	0.71 (0.17–1.95)	0.573	1.57 (1.14–2.13)	0.005
Injury-related characteristics				
Work-related injury				
No	Ref		Ref	
Yes	0.90 (0.51–1.66)	0.724	0.74 (0.56–0.98)	0.034
Injury severity (NISS)				
1–3	Ref		Ref	
4–6	0.65 (0.34–1.22)	0.192	0.73 (0.54–0.98)	0.035
≥ 7	1.15 (0.46–2.51)	0.751	0.97 (0.61–1.48)	0.887
Hospitalisation				
No	Ref		Ref	
Yes	0.31 (0.11–0.71)	0.013	0.72 (0.50–1.01)	0.065
Perceived threat to life				
No	Ref		Ref	
Yes	1.91 (0.90–3.68)	0.067	1.69 (1.13–2.44)	0.007
Perceived threat of long-term disability				
No	Ref		Ref	
Yes	1.09 (0.61–1.91)	0.770	1.18 (0.90–1.56)	0.227
Trouble accessing healthcare services				
No	Ref		Ref	
Yes	1.06 (0.40–2.31)	0.892	1.35 (0.90–1.96)	0.126

**Table 3:** Multivariable analyses—adjusted relative risk of characteristics and disability 12 years post-injury among migrants

Migrant characteristics	Adjusted relative risk^a^ (95% CI)
Paid employment	
No	Ref
Yes	0.43 (0.16–1.42)
Household income	
Adequate	Ref
Inadequate	2.08 (1.09–4.03)
Living arrangements	
Alone	Ref
With family	0.28 (0.11–0.81)
With non-family	0.53 (0.18–1.64)
Injury severity (NISS)	
1–3	Ref
4–6	1.02 (0.49–2.07)
≥ 7	1.93 (0.68–4.81)
Disability (WHODAS II)	
No/lesser (0–9) Considerable (≥ 10)	Ref 1.94 (0.60–5.25)
Hospitalisation	0.18 (0.04–0.55)
Perceived threat to life	2.93 (1.17–6.69)

^a^All variables adjusted for other variables in the model

A combination of directed acyclic graphs (DAGs) and a purposeful selection of covariates was used to develop initial multiple regression models [25, 26]. Full models were populated with all significant predictors (*p* < 0.1) from the univariate models and the minimal sufficient adjustment set identified from DAGs [27], and backwards elimination using Akaike’s Information Criterion (AIC) was used to help select the final model [28, 29]. We calculated adjusted relative risks (a*RR*) and their respective 95% confidence intervals (CI) for each included predictor in the multivariable models. All analyses were performed in R [30].

## Results

Of 2856 POIS participants interviewed 3 months post-injury, 2256 participated in the 24-month follow-up interview (Supplementary Fig. 1) [2]. For the 12-year follow-up, we interviewed 1543 participants, of whom 1497 had WHODAS II and migrant status data available. The majority (79.9%) of participants 12 years post-injury were non-migrants (*n* = 1196); 20% were migrants (*n* = 301).

There were no differences in gender between migrant and non-migrant groups (Table 1). There was a significant difference with regards to their age (*p* = 0.017), likely resulting from a larger proportion of the migrants aged 55–64 years than for the non-migrants. A larger proportion of migrants reported having a post-secondary education than non-migrants (*p* < 0.001). Though migrants and non-migrants were just as likely to report paid employment, a lower proportion of migrants reported adequate income pre-injury (*p* = 0.041). A larger proportion of migrants reported higher levels of comfort in faith/spiritual beliefs and non-smoking/-alcohol/-recreational drug use compared to non-migrants; a larger proportion of non-migrants had higher NISS scores (4 +) than migrants.

### Univariate Analysis: Pre-injury Socio-demographic Characteristics

At 12 years post-injury, 16.3% of migrants (*n* = 49) and 17.7% of non-migrants (*n* = 212) reported considerable disability. Migrants who reported an inadequate pre-injury household income had a nearly three-fold higher risk of having considerable disability 12 years post-injury than migrants who reported an adequate income (RR 2.74; 95% CI 1.55–4.96) (Table 2). Similarly, non-migrants with an inadequate pre-injury income had a significantly higher risk of having a disability 12 years post-injury than non-migrants with an adequate income (RR 1.46; 95% CI 1.10–1.92). Additionally, non-migrant males had lower risk of disability 12 years post-injury (RR 0.77; 95% CI 0.59–1.01). Older age at time of injury, compared to 18–24 year-olds, was also related to risk of disability 12 years post-injury among non-migrants (35–44 year-olds RR 1.78; 95% CI 1.04–3.26 and 45–54 year-olds RR 1.82; 95% CI 1.08–3.30), but this increased risk of disability was not observed for 55–64 year olds (*p* = 0.263). Non-migrants with any formal education had a lower risk of disability than non-migrants with no formal education (post-secondary RR 0.55; 95% CI 0.40–0.77, and secondary RR 0.51; 95% CI 0.34–0.77). Non-migrants in paid employment had a lower risk of disability than non-migrants not in paid employment (RR 0.56; 95% CI 0.40–0.85). Those non-migrants with a small or very small family involvement were less likely to have a disability at 12 years post-injury than those with large family involvement (RR 0.67; 95% CI 0.48–0.98). Lastly, non-migrants who reported comfort in faith or spiritual beliefs had a significantly higher risk of disability at 12 years than those who reported no comfort (RR 1.61; 95% CI 1.14–2.29).

### Univariate Analysis: Pre-injury Health-Related Characteristics

Migrants with considerable pre-injury disability were more likely to have considerable disability at 12 years, than migrants with lesser/no pre-injury disability (RR 4.93; 95% CI 1.70–11.34). Similarly, non-migrants with considerable pre-injury disability were more likely to have considerable disability at 12 years than non-migrants with lesser/no pre-injury disability (RR 2.81; 95% CI 1.85–4.10). Additionally, non-migrants with poor health pre-injury had significantly increased risk for disability at 12 years than non-migrants with better health (RR 3.23; 95% CI 2.15–4.67). Non-migrants who self-reported smoking (RR 1.57; 95% CI 1.14–2.13) or recreational drug use (RR 1.74; 95% CI 1.31–2.30) had higher risks of disability at 12 years than non-migrants not reporting these uses. Lastly, non-migrants who reported alcohol use before the injury had lower risk of disability at 12 years follow-up (RR 0.50; 95% CI 0.35–0.73).

### Univariate Analysis: Injury-Related Characteristics

Among non-migrants, those with a work-related injury, or experiencing an injury categorised as moderately severe, were less likely to experience considerable disability 12 years post-injury than non-migrants with injuries that were not work-related (RR 0.74; 95% CI 0.56–0.98) or whose injury was of mild severity (RR 0.73; 95% CI 0.54–0.98). Migrants hospitalised for injury were less likely to have considerable disability 12 years post-injury than non-hospitalised migrants (RR 0.31; 95% CI 0.11–0.71)*.* Similarly, hospitalised non-migrants were significantly less likely to have considerable disability 12 years post-injury than non-migrants who were not hospitalised (RR 0.72; 95% CI 0.50–1.01). Migrants and non-migrants alike, who perceived their injuries as a threat to their life at the time of the injury, had an increased risk of considerable disability 12 years post-injury (RR 1.91; 95% CI 0.90–3.68 and RR 1.69; 95% CI 1.13–2.44, respectively).

### Primary Analysis: Multiple Regression Identifying Predictors of Disability for Migrants 12 Years Post-injury

After adjusting for all other predictors in the multivariable model, migrants reporting an inadequate household income pre-injury had an increased risk of disability 12 years post-injury (aRR 2.08; 95% CI 1.09–4.03) (Table 3). Migrants had a reduced risk of disability 12 years post-injury when living with family members compared to those living alone, and when hospitalised for injury compared to those not hospitalised (aRR 0.28; 95% CI 0.11–0.81 and aRR 0.18; 95%CI0.04–0.55 respectively). However,. migrants who perceived the injury as a threat to their life were significantly more likely to have considerable disability 12 years post-injury (aRR 2.93; 95% CI 1.17–6.69). The *R*^2^ value of the final model of the migrant multivariable analysis was 0.23. Additionally, we observed no multicollinearity between included predictors.

### Secondary Analysis: Multivariable Models Identifying Predictors of Disability 12-Years Post-injury for All POIS Participants (Migrants and Non-migrants)

In multivariable analyses, we found migrants had similar risks of long-term disability 12 years post-injury as non-migrants after adjustment for potential confounders (aRR 1.05; 95%CI 0.73–1.49) (Supplementary Table 2).

## Discussion

In this longitudinal cohort study of injured adults in NZ with an ACC injury entitlement claim (between 2007 and 2009), we found 16% of migrants reported considerable disability 12 years post-injury. Univariable analyses found pre-injury inadequate household income and pre-injury disability were associated with an increased risk of considerable disability 12 years post-injury for migrants; whereas, being hospitalised for the injury was associated with a reduced risk of long-term disability. These associations were also found among non-migrants, although other characteristics were also associated with considerable disability among non-migrants. Multivariable analyses found migrants with an inadequate pre-injury household income (aRR 2.08; 95% CI 1.09–4.03) or perceiving a threat to their life at the time of injury (aRR 2.93; 95% CI 1.17–6.69) were significantly more likely to experience considerable disability 12 years post-injury compared to migrants without those characteristics. Conversely, migrants living with family were significantly less likely to experience considerable disability 12 years post-injury compared to migrants living alone (aRR 0.28; 95% CI 0.11–0.81), and hospitalised migrants had a lower risk of disability (aRR 0.18; 95% CI 0.04–0.55) compared to non-hospitalised migrants.

There was a tendency for those experiencing pre-injury disability to be at increased risk of disability 12 years post-injury, although this was not statistically significant (aRR 1.94; 95%CI 0.60–5.25). This tendency was also found in the earlier analyses looking at predictors of considerable disability at 3- and 24-months post-injury [2]. However, two characteristics, proximate to the injury event itself, were strongly (and independently) associated with long-term disability—having an inadequate pre-injury household income and perceiving a threat to their life at the time of the injury. These characteristics could be identified early in the post-injury pathway, allowing for opportunities to intervene and potentially improve long-term disability outcomes for migrants with these characteristics.

Earlier analyses found injury severity and perceived threat of disability at the time of injury to be associated with disability at 3 months post-injury (but not at 24 months), and comorbidities to be associated with disability at both 3- and 24-months post-injury [2]. Of these earlier associated characteristics, only NISS was retained in our final 12-year multivariable model—and was not significantly associated with disability (beyond a tendency for those with NISS ≥ 7 to be at increased risk).

Of migrants who reported a threat to life, 23% had NISS scores ≥ 7; among migrants who reported no perceived threat to life, 10% had NISS scores ≥ 7. NISS scores are based on immediate anatomical injury severity [31]—and not the likelihood of long-term outcomes[32]. Our question, developed for POIS, asking respondents about the self-perceived severity of their injury having such a strong association (aRR 2.93) with long-term disability outcomes for migrants merits further investigation in other injury outcome studies, and for other specific outcomes. Elsewhere, self-reported responses to brief questions have been found to be strongly associated with outcomes such as mortality [33].

Interestingly, we found hospitalisation for injury was related to a markedly lower risk of long-term disability among migrants. Among all participants, being hospitalised for injury had significantly lower risk of disability at 12 years post injury independent of migrant status; similar to previously reported findings at 24 months post-injury [19]. We hypothesise that this finding for migrants specifically, may result from hospitalised migrants receiving timely and maximal supports from health providers and ACC—whereas non-hospitalised migrants may experience delays in access to treatment or support with negative effects for their long-term disability outcome. Further research is warranted to better understand this relationship.

Inequities in healthcare access between migrants and non-migrants have a multifaceted effect on outcomes. Migrants can experience the burden of worsening health inequities resulting from the cumulative effects of lower household incomes, chronic conditions, and decreasing health over time compared to non-migrants [34, 35]. Earlier POIS analyses found 45% of injured migrants, and 42% of non-migrants, experienced considerable disability three months post-injury [2]. By 24 months post-injury, the proportion of participants who experienced disability had reduced to 13% for both groups—albeit still considerably higher than pre-injury proportions (4% for migrants and 6% for non-migrants) [2]. In the current analysis, we found the proportions experiencing considerable disability 12 years post-injury increased slightly from 24 months—but remain similar between migrants and non-migrants; migrants were not found to be at increased risk of long-term disability compared to non-migrants (aRR 1.05; 95%CI 0.73–1.49; Supplemental Table 2). It is important to note that the proportion of migrants experiencing disability (16.3%) remains higher than pre-injury; identifying opportunities for reducing long-term post-injury disability seem warranted.

When considering both migrants and non-migrants (Supplemental Table 2), we found that, after adjusting for migrant status, several sociodemographic characteristics, pre-injury disability and health characteristics were associated with long-term disability. Males were significantly less likely to have considerable disability 12 years post-injury than females. Of note, males and females had similar initial injury severity. Prior analyses of this cohort have shown that males also have reduced risk of disability at 24 months [2]. Importantly, we found males were significantly more likely to be lost to follow-up, so selection bias cannot be ruled out as at least partially accounting for this relationship between sex and disability at 12 years. We found that participants who reported a post-secondary education were less likely to have a disability at 12 years follow-up than those with no formal education. This finding should be considered in the context of education levels of migrants and non-migrants. In this cohort non-migrants, on average, have a higher proportion with no formal education (15%) than migrants (5%), which is in contrast to studies of migrant workforces in other countries that have found migrants often have less formal education than non-migrants [36], though this is largely dependent on the type of employment migrants are engaged in.

We note a number of limitations to our study. Our models had poor predictive abilities with low *R*^2^ values for 12-year disability outcomes for migrants specifically (0.23), suggesting much of the variance in these outcomes is unexplained by our models. Ultimately, if our goal was to make predictions of disability, having more participants in the cohort (or unmeasured predictors to our models) may have been necessary to derive better predictive results. However, our goal was to understand factors with significant impact on disability at 12-years post injury and identify avenues of possible intervention for injured participants, so models with lower predictability may be of less consequence. Further, we consider our multivariable models somewhat robust as the overall estimates, particularly among the retained predictors, changed little after removing highly influential observations in a sensitivity analysis (Supplemental Table 1). Additionally, we found that our cohort 12 years post-injury differed from those who were lost to follow-up significantly and may have introduced bias to our analyses. However, our findings at 12 years are largely reflective of the findings from previous analyses at 3- and 24-months post-injury, suggesting the bias introduced over time due to loss to follow-up may be of little consequence to our overarching findings. Lastly, the larger sample size for non-migrants (*n* = 1196) allowed for more power to identify predictors of disability relative to the power for migrants (*n* = 301).

### Supplementary Information

Below is the link to the electronic supplementary material.

Supplementary file1 (DOCX 152 kb)
