# Supplementary material for: Long-Term Disability Outcomes for Migrants (and Non-migrants) 12 Years Post-injury: Results from the Prospective Outcomes of Injury Study in New Zealand
Source: J Immigr Minor Health. 2023 Aug 5;25(6):1354–62. doi: 10.1007/s10903-023-01526-w (PMC10632225; doi:10.1007/s10903-023-01526-w)
Supplement: Supplementary file 1 — Supplementary file1 (DOCX 152 kb) [file 10903_2023_1526_MOESM1_ESM.docx]

**Supplemental Materials**

*Supplemental Figure 1: POIS Participant Recruitment*


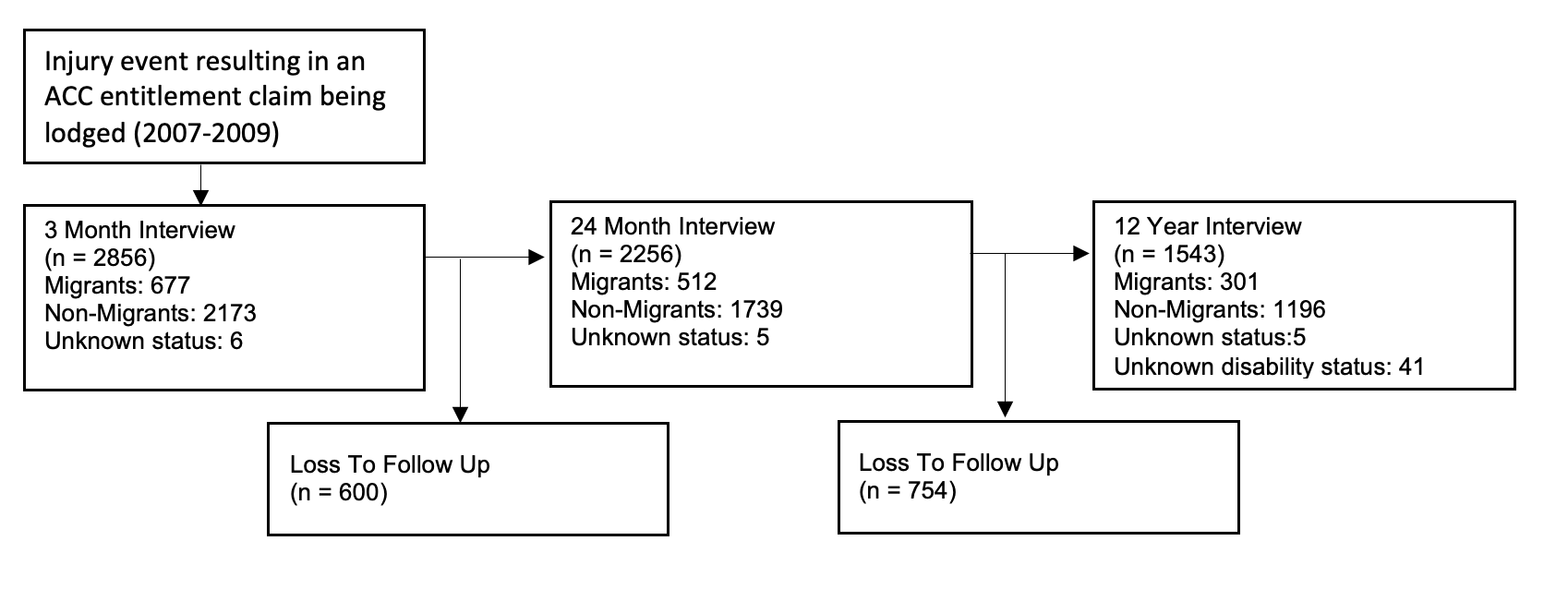


*Supplemental Table 1: Sensitivity Analyses—Adjusted Relative Risk of characteristics and disability among migrant 12 years post-injury*

| *Characteristic* | *Adjusted Relative Risk^1^ (95% CI)* |
| --- | --- |
| *Household income*  Adequate  Inadequate | *Ref*  *2.58 (1.34-5.12)* |
| *Living arrangements*  Alone  With family  With non-family | *Ref*  *0.21 (0.08-0.59)*  *0.40 (0.14-1.23)* |
| *Injury severity (NISS)*  1-3  4-6  ≥7 | *Ref*  *1.01 (0.47-2.07)*  *2.47 (0.86-6.17)* |
| *Disability (WHODAS II)*  No/lesser (0-9)  Considerable (≥10) | *Ref*  *3.79 (1.23-9.60)* |
| *Hospitalisation* | *0.06 (0.00-0.30)* |
| *Perceived threat to life* | *2.40 (0.92-5.55)* |

1. *All variables adjusted for other variables in the model*

*Supplemental Table 2: Multivariable analyses (secondary analysis)—Adjusted Relative Risk of characteristics and disability at 12 years post-injury Post-Injury*

| *Characteristic* | *Adjusted Relative Risk^1^ (95% CI)* |
| --- | --- |
| *Migrant status*  Non-migrant  Migrant | *Ref*  *1.05 (0.73-1.49)* |
| *Gender*  Female  Male | *Ref*  *0.74 (0.55-0.98)* |
| *Highest education qualification*  No formal  Post-secondary school  Secondary school | *Ref*  *0.65 (0.46-0.95)*  *0.77 (0.51-1.17)* |
| *Paid employment* | *0.69 (0.46-1.10)* |
| *Household income*  Adequate  Inadequate | *Ref*  *1.32 (1.00-1.76)* |
| *Comfort in faith or spiritual beliefs*  Not at all  Very much/quite a bit  Somewhat/A little bit | *Ref*  *1.67 (1.17-2.40)*  *1.36 (0.95-1.95)* |
| *Family involvement*  Very large/Large  Small/Very small | *Ref*  *0.68 (0.48-1.00)* |
| *General health*  Not poor health  Poor health | *Ref*  *2.21 (1.39-3.40)* |
| *Disability (WHODAS II)*  No/lesser (0-9)  Considerable (≥10) | *Ref*  *1.81 (1.13-2.82)* |
| *Smoking* | *1.38 (1.00-1.87)* |
| *Regular alcohol use pre-injury* | *0.64 (0.45-0.94)* |
| *Recreational drug use* | *1.46 (1.01-2.07)* |
| *Work-related injury* | *0.80 (0.60-1.08)* |
| *Hospitalisation* | *0.63 (0.42-0.90)* |
| *Perceived threat to life* | *1.38 (0.92-1.99)* |

1. *All variables adjusted for other variables in the model*
